# The Case of “Frederick the Noble”

**Published:** 1888-11

**Authors:** 


### THE CASE OF "FREDERICK THE NOBLE."

A brief mention was made in the December number of this journal, 1887, of the treatment of the case of the dying Crown Prince and afterward, for a few months, Emperor of Germany. The whole civilized world watched with the keenest anxiety the royal prisoner, Frederick the Noble, bound in his palace with the fetters of disease and death. In the hands of Sir Morrell Mackenzie, a distinguished specialist of London, and of German physicians of like renown, before and since his death, an unnecessary dispute has arisen in regard to the management and treatment of the case, which has recently been intensified by some articles from the pens of the principal physicians attending him from both nations. The following from Dr. J. Solis Cohen, an American of great distinction as a throat specialist, is a fair and impartial resumé of the whole case, and we feel assured will be perused with interest by the readers of this journal. We are indebted for the article appended to the New York *Herald*, which gave to the world the advance sheets of Dr. Mackenzie's book on the same case a day in advance of all others:

"Dr. J. Solis Cohen, who is known in Europe as well as America as an eminent and successful specialist in throat diseases, has refrained till now from expressions of opinions on the treatment of the German Emperor. 'I did so,' he said yesterday, 'from a sense of propriety, because the character of the public telegrams was not such as to justify an opinion in the absence of accurate information, and because I deemed it an impertinence toward those in charge of the case and to the family.'

"The records of Professor Von Bergmann and of Professor Gerhardt vary so much from that of Sir Morrell Mackenzie that the questions between them are largely questions of veracity—questions which cannot be decided on the American side of the Atlantic, where all of them are held in equal honor and in equal professional repute. If one account be true the other must be inaccurate.

"Gerhardt accuses Mackenzie of a slip in manipulation, attributable to a want of due illumination, in which he tore out a piece of the healthy vocal band instead of the diseased one—a charge that is incredible, and which is repudiated by Mackenzie in no honeyed terms.

"That Gerhardt and Tobold were correct in their diagnosis of cancer before ever Mackenzie appeared upon the scene has been only too fully proven by the unhappy sequel. Despite puerile attempts to disparage the reputation, skill and experience of these gentlemen, I feel certain that their professional brethren generally have as much confidence in them as in any other laryngoscopist in the whole of Europe or elsewhere. I have this confidence.

NEGATIVE EVIDENCE IS NO PROOF.

"Mackenzie, on the other hand, was just as correct when he declined to recommend an operation by external access until after an attempt had been made to secure a fragment of the growth for microscopic examination. The result of the examinations in this case shows only too plainly that while the positive evidences of microscopic inspection are of the greatest utility, negative evidence is no proof to the contrary of a proposition, and that too great confidence in the same may lead us fatally astray. Mackenzie justified by his manipulations his confidence in the use of forceps to remove a portion of the growth.

"After the first portion of the growth had been removed and pronounced non-malignant, the rumor extended that the patient had been saved from a projected sacrifice of at least one-half of his larynx, all preparations for which had been already made, with the intention of its execution the morning after Mackenzie's arrival. Von Bergmann denies this in the strongest language.

All that was at all under contemplation at the time, he asserts, was to open into the parts from the outside, and then remove the little growth thoroughly, together with a portion of the vocal band upon which it had been developed, so as to get rid of the disease bodily and at once with the least danger.

"This operation he had performed successfully two years before upon a man whose larynx, he says, he vainly requested Mackenzie to come and examine. He was anxious to exhibit the condition of the interior, which, he says, resembled a picture re-produced by me from the larynx of a gentleman from whom, more than twenty years ago, I had successfully removed a cancer by laryngotomy, the very same operation which he had recommended their august patient also '*dieselbe operation die ich be unserem hohen Patienten vorgeschlagen hatte*'. This operation, in my opinion the result of mature experience, would not have endangered life any more than a tracheotomy, perhaps not as much.

"In this opinion I am in accord with Von Bergmann. In the light of subsequent developments, I have no doubt that it would have been the very best and the very safest procedure at that time. Whether the ultimate result would have been better than that which followed the course pursued, mortal man has no right to claim knowledge. It would hardly have been worse; it might have been salvation.

MACKENZIE RIGHT THIS TIME.

"Another complaint against Sir Morrell Mackenzie is a failure to fulfill a solemn promise to cease his manipulations and refer the patient to the German physicians as soon as it became evident that his procedures were not arresting the progress of the disease, to the end that radical measures might be instituted at a date in its clinical progress sufficiently early to hold out a reasonable prospect of success.

"As is well known, the case progressed until it became evident that death would ensue despite any operation, and then the radical operation of removal of the larynx was very justly repudiated by Mackenzie and by the patient at that late date. The time for such procedure had passed. It is quite possible that this time was allowed to lapse with the cognizance of the Crown Prince, who may have made up his mind long before not to run the great immediate risk attached to it under any circumstances. In a study made by me some years ago as to whether the operation of excision of the larynx tended to increase the average tenure of life, and w[illegible]l known to the profession, it was shown that very many more years of life were cut off from the remnants of existence insured to the whole number than were added to the few who did not die from the operation itself, or from speedy recurrence of the disease; and although cases have been selected since with greater precaution, it is still evident, and, I fear, will long remain so, that the greatest good to the greatest number is practiced by avoiding the operation, except in a very limited, special class of cases in which the risk to the individuals is justified.

"Why Mackenzie did not perform tracheotomy himself I have always been at a loss to determine. I certainly would not allow another to perform such an important operation on a patient of mine, unless I felt that he was the safer operator, and then if he should cut a trifle to one side, I would feel that I might have done worse. According to the accounts of the German physicians Bramann operated under very trying and irritating circumstances.

THAT CANULA DISCREPANCY.

"The greatest discrepancy in the accounts is in reference to the failures of Von Bergmann to introduce a canula into the trachea of the patient when he was suffocating. Von Bergmann states that, on April 12, he received an urgent note from Mackenzie, reading thus:

'DEAR PROFESSOR VON BERGMANN—We have difficulties with the canula, and I shall be glad if you will see the Emperor with me as soon as possible.

Yours truly, MORRELL MACKENZIE.'

"This is the note of which Sir Morrell Mackenzie writes. 'It is no exaggeration to say that these hastily scribbled lines proved to be the death warrant of the Emperor.'

"Compare the picture of the Emperor's condition on their entrance into his room. Mackenzie says that 'they found the Emperor engaged in writing; that the inspiration was distinctly audible, but beyond this there was not the slightest indication of any difficulty in breathing.' Von Bergmann says that 'he turned with Mackenzie to the Emperor and was shocked to find him sitting in a chair in a state of suffocation (*auf einem Stuhle silzend im Ersticken*).' The cheeks and lips were blue; there was a stridor in inspiration which could be heard in the adjoining room, the inspiration being of the most laborious character, with distention of all his muscles and with retraction of the soft tissues below the breast bone, as distinctly seen through his open coat. He says that the outer canula was not within the windpipe, but only reached it, and that Mackenzie told him that several attempts had been fruitlessly made since morning to introduce the inner canula.

"It may be stated here that the inner canula reaches a little further than the outer one, so as to leave an unencumbered passage for air through the latter when the former is removed for purposes of cleaning. If now the end of the outer canula did not reach the windpipe, but lay choked up against it, it is comprehensible how the inner canula could not be got any further.

"Von Bergmann gives a very graphic account of his attempts to introduce a canula, which are not at all in accord with the horrid recital detailed by Mackenzie.

MACKENZIE WRONG IN THIS.

"One other phase of the controversy deserves mention on account of its influence on the public. That is the possibility of transforming an innocent growth into a cancer by manipulation. This point has been raised by Sir Morrell Mackenzie. For many years I believed this possible. I believed it to have taken place under my own hands in the very patient to whom allusion has been made as a living example of more than twenty years immunity after an operation by external access. I believed it to have taken place in a number of cancers of the larynx transferred to my care in a late stage of their history after submission to va[illegible]ous intralaryngeal procedures. In my professional treaties, the[illegible]efore, I called attention to this belief. Riper judgment, and the result of an investigation now under way in London, and well known to laryngologists throughout the world, show that this is not the case, and that with very few exceptions—so few, indeed, that they may be almost practically discarded—such apparent transformations occur only in cases malignant from the outset, such as has been that of the Emperor of Germany, and in which the intralaryngeal procedures have followed a mistake in diagnosis.

"It is therefore a matter of surprise to me to see so fair and critical a man as Sir Morrell Mackenzie attribute possible transformation of the kind, even by imputation, to the electro-caustic application by Gerhardt. It might just as well have proceeded from Mackenzie's own manipulations with forceps and with electric cautery. I do not believe that any such effect was produced in either instance, and I cannot think that Mackenzie believes it. I take this occasion to express this opinion because this passage, in the widespread dissemination of Mackenzie's book, will have the influence to deter patients with innocent growths from having them extirpated lest they turn into cancers, and thus condemn themselves practically to self-sacrifice.

FREDERICK KNEW HIS MAN.

"Finally, if we are to believe the cabled reports of the daily press from time to time during the clinical history of the unfortunate Emperor's case, Mackenzie is intitled to the greatest credit for the promptness and efficacy with which every emergency was met, often under the most trying circumstances. Not the least important truth of his ability is the tenacity with which his distinguished patient clung to his ministrations, despite strong inducements to the contrary. That so intelligent a patient as this Emperor was, with so much to live for, should be mistaken in the character of the services of his physician is beyond conception, and an imputation of the kind evinces a want of respect for his judgment and good sense which is disgraceful to his memory.

"If for special reasons Sir Morrell Mackenzie so expressed himself at times late in the case as to imply a doubt as to his knowledge of the true nature of the malady, we may rest assured that he did so with the full consent of his patient, and not improbably at his very suggestion."

